# Updates on Interventional Radiology in Sudan: Training, Practice, and Students’ Awareness and Interest, a University of Khartoum Perspective

**DOI:** 10.7759/cureus.89624

**Published:** 2025-08-08

**Authors:** Shafee S Almahi, Mohamed H Fadul, Moayad A Elgassim, Soudad A Shila, Ahmed Ali, Abdalla Fadul

**Affiliations:** 1 Faculty of Medicine, University of Khartoum, Khartoum, SDN; 2 Department of Medical Education, Hamad Medical Corporation, Doha, QAT; 3 Faculty of Medicine, University of Medical Sciences and Technology, Khartoum, SDN; 4 Internal Medicine Department, Hamad Medical Corporation, Doha, QAT; 5 Geriatrics and Long-term Care, Hamad Medical Corporation, Doha, QAT

**Keywords:** interventional radiology, medical education, medical students, radiology, sudan

## Abstract

Background: Interventional radiology (IR) is a growing field with wide applications. However, awareness of IR is limited, especially among medical students and particularly in developing countries where its practice is deficient.

Purpose: To assess and compare knowledge, awareness, and interest in IR among medical students at the University of Khartoum, Sudan, a developing country where the practice of IR faces many challenges, including the 2023 war. It also provides an update regarding IR practice and training in Sudan and a review of relevant literature, and serves to improve awareness and interest in IR.

Methods: A cross-sectional facility-based study was conducted. Participants were selected using simple random sampling. A multi-section online questionnaire assessed the subjective and objective knowledge about IR and the participants’ interest and views regarding its introduction to the undergraduate program. Data were analyzed using Excel (Microsoft Office) and Statistical Package for Social Sciences (SPSS, IBM).

Results: One hundred eighty-one students were included. 59.7% were females, and 60.2% were in preclinical years. 75.1% subjectively reported poor or no knowledge about IR. A total of 71.8% suggested mixed surgical and radiological IR training. Students’ awareness of IR names, advantages, procedures, and roles is reported. Students' knowledge and awareness were found to be lacking. Only 13.8% were considering a career in IR.

Conclusion: IR practice in Sudan is deficient, especially after the war. Although their awareness was inadequate, students showed an interest in learning more about IR. IR knowledge and exposure need to be improved, but the introduction of IR and other advanced subspecialties to undergraduate curricula requires more research and attention by medical education specialists.

## Introduction

Interventional radiology (IR) is a rapidly advancing specialty within modern medicine that has emerged from angiography but with additional novel therapeutic applications [1]. In 1963, Dr. Charles T. Dotter used Seldinger’s catheterization technique for performing intravascular surgery. In 1964, he published his novel article, which demonstrated the use of serial dilators to widen an atherosclerotic femoral artery in a patient who had come for amputation and returned with a salvaged limb [2]. It was not until the mid-1970s that trans-catheter therapeutic procedures became common in the United States [1]. IR techniques have transformed the treatment of malignant and benign conditions in elective and emergency settings by enabling a wide range of minimally invasive life-saving or quality-of-life-improving procedures [3]. IR procedures are categorized into vascular (vessel opening or closing), non-vascular, neurovascular, and oncological [4]. Alongside their clinical roles (outpatient clinics, inpatient admissions, procedures, etc.), interventional radiologists are involved in other duties such as medical education and lectures and multidisciplinary meetings [5, 6].

Unsurprisingly, the popularity of IR procedures has grown in recent years, and they are getting more favored because, compared to their surgical counterparts, IR procedures shorten hospital length of stay, decrease morbidity and mortality, improve survival outcomes, and lessen postoperative complications [3]. Some IR procedures that are recognized to be safe and effective or less morbid than their surgical counterparts include vessel embolization in uterine fibroids and benign prostatic hyperplasia, revascularization in peripheral vascular disease and stroke, biliary drainage, fallopian tube recanalization, thermal or cryoablation of focal tumors, chemo- or radio-embolization of liver primaries or metastases, and bland embolization of vascular tumors [7-13].

Most IR training programs consist of 5-7 years and follow a classical pathway of diagnostic radiology (DR) followed by IR training. In the United Kingdom, the Royal College of Radiologists mandates three years of DR followed by three years of IR training. The training comprises five years of DR followed by one year of IR in Canada, three years of DR followed by two years of IR in Thailand, and 1-3 years of independent IR training in Malaysia. In the United States, there are two alternative programs: the integrated IR residency and the independent IR residency with early specialization in IR (ESIR), which also includes DR training before the IR training [14-18]. There is no IR training program in Sudan, and all registered interventional radiologists were trained in Malaysia, Jordan, or Thailand. However, DR training in Sudan started in 1992 at the University of Khartoum and was later adopted by the Sudan Medical Specialization Board (SMSB) in 2003, training up to 35 diagnostic radiologists every year [19].

In Sudan, IR services were available only in Khartoum before the 15th of April 2023 war, and recently in El-Obeid; therefore, the lack of these services represents a real issue, especially in rural areas. IR procedures are typically performed in dedicated, equipped IR suites. There were three of them before the war in Sudan. The first two suites, launched in 2016, were private. The third governmental one was established at Suba University Hospital (SUH), affiliated with the University of Khartoum, in 2021 and is the only vascular IR suite. Consequently, IR procedures are also done in fluoroscopic rooms or shared angiography or cardiac catheterization units. Moreover, only four of the seven SMSB-registered interventional radiologists continued their work in Sudan. Therefore, many IR procedures, including drainage, biopsies, and insertion of percutaneous nephrostomy tubes and dialysis catheters, are handled by diagnostic radiologists, urologists, and vascular surgeons [19, 20]. The challenges for IR services in Sudan include a lack of professional awareness among healthcare workers, the limited availability of trained interventional radiologists and IR suites, the logistic, infrastructural, and financial challenges, the unsteady political situation, and, more recently, the 2023 war which has largely overwhelmed the healthcare system and medical education all over the country [20].

The Faculty of Medicine at the University of Khartoum is Sudan’s first and most prestigious medical school, founded in 1924. Its accredited academic curriculum comprises three preclinical and three clinical years. The main radiology course is provided in the eighth semester, besides other dedicated radiology lectures within anatomy and neurosciences courses in the preclinical semesters. As mentioned, DR training in Sudan was first started at this faculty, and the only governmental IR suite in Sudan (SUH) is also affiliated with it. Several recent international and regional surveys revealed a lack of knowledge and exposure to IR among many undergraduate programs [21-27]. In parallel, another study showed that most medical students were aware of less than 50% of the common IR procedures, echoing similar results of insufficient undergraduate IR exposure [28].

From an IR standpoint, for the patients and the healthcare system to benefit from minimally invasive procedures, all doctors, including future ones, must be well aware of IR and its applications [6]. As an illustration, according to the Royal College of Radiologists, there was a 23% shortage of radiologists in 2018, and it is expected to rise. This is owing to the limited undergraduate IR teaching and lack of exposure and awareness of the specialty among medical trainees [28-30]. These assumptions were supported by previous studies that found that the early introduction of IR courses enhanced awareness and interest in IR among medical students [31-33].

The purpose of this study was to assess and compare knowledge, awareness, and interest in IR among medical students at the top medical school in Sudan, a developing country where the practice of this field faces many challenges, including the war. In addition, it also provides an update regarding the practice and training of IR in Sudan and a review of the relevant literature, and serves to improve awareness and interest in IR among the students.

## Materials and methods

This descriptive cross-sectional study was conducted at the Faculty of Medicine, University of Khartoum, Sudan, in December 2023. The study population was all current students. With a 90% confidence level and a 5% margin of error, the calculated sample size was 240. Participants were selected using simple random sampling. Data were collected through an online survey using a multi-section self-administered Google Forms questionnaire. With a response rate of about 76%, 181 students participated in the study.

The questionnaire contained questions on the participant’s age, sex, academic level, and subjective and objective knowledge about IR, including its names, procedures, applications, advantages, clinical training, and duties. It also contained questions on participants’ interest in learning more about the discipline, the introduction of IR to undergraduate studies, and the suitable learning methods. Finally, participants were asked whether they would consider an IR career according to their current knowledge and perception of the specialty. The questionnaire was developed by the authors after reviewing the literature for similar studies and questionnaires (see Appendices). Permission was taken from the authors to use parts of their questionnaires. A small pilot study evaluated the questionnaire for any issues.

Data was cleaned, managed, and analyzed using Microsoft Office - Excel 2016 (Microsoft Corp., Redmond, WA, USA) and the Statistical Package for Social Sciences (SPSS) version 26 (IBM Corp., Armonk, NY, USA). The characteristics of the study population were described in means, standard deviations, frequencies, and percentages, and illustrated in graphs. Suitable graphs were used to report the participants’ knowledge and awareness of the above-mentioned aspects. The questionnaire was anonymous. Informed consent was taken from every participant.

## Results

The study included 181 students. 59.7% (n=108) of them were females. 60.2% (n=109) of them were in their preclinical years (the first, second, and third years) and 39.8% (n=72) were in their clinical years (the fourth, fifth, and sixth years). The most common age group was 21-25 years (53.6%, n=97). The participants’ characteristics are described in Table 1.

**Table 1:** Characteristics of the study participants (n=181) Data is represented in frequencies (N) and percentages (%).

Characteristic	Frequency (Percentage)
Age Group	
17 - 20 years	77 (42.5%)
21 - 25 years	97 (53.6%)
26 - 30 years	7 (3.9%)
>/= 31 years	0 (0%)
Sex	
Female	108 (59.7%)
Male	73 (40.3%)
Year Level	
Preclinical	109 (60.2%)
Clinical	72 (39.8%)

Students were asked about their subjective knowledge of IR. Only a minority of them (3.9%, n=7) subjectively described their knowledge of IR as “Good”, while most of them reported “Poor” knowledge (41.4%, n=75) or “None” at all (33.7%, n=61).

**Figure 1:**
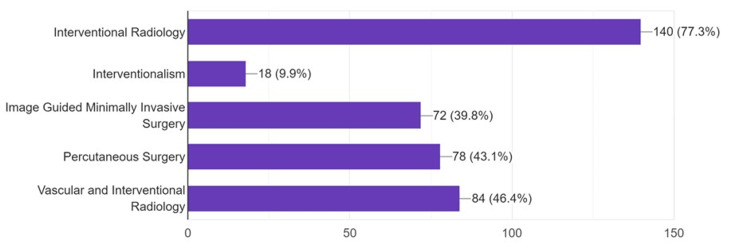
Students’ opinion on other names used to refer to interventional radiology (n=181) Data is represented as frequencies (N) and percentages (%).

Students were asked about names or terms that are used to refer to the IR specialty. They showed less awareness of the other correct names, such as Image-Guided Minimally Invasive Surgery (IGMIS; 39.8%, n=72) (Figure 1).

**Figure 2:**
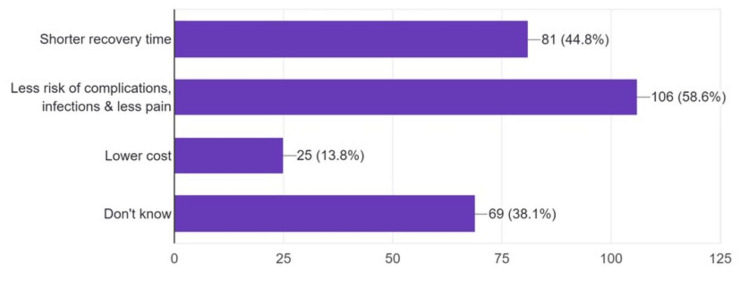
Students’ awareness of the advantages of interventional radiology over conventional surgery (n=181) Data is represented as frequencies (N) and percentages (%).

Regarding postgraduate training of interventional radiologists, 71.8% (n=130) of the students suggested a mixed surgical and radiological postgraduate training program. Students were aware of some IR advantages over traditional surgery, but only 13.8% (n=25) thought it might generally cost less (Figure 2).

**Figure 3:**
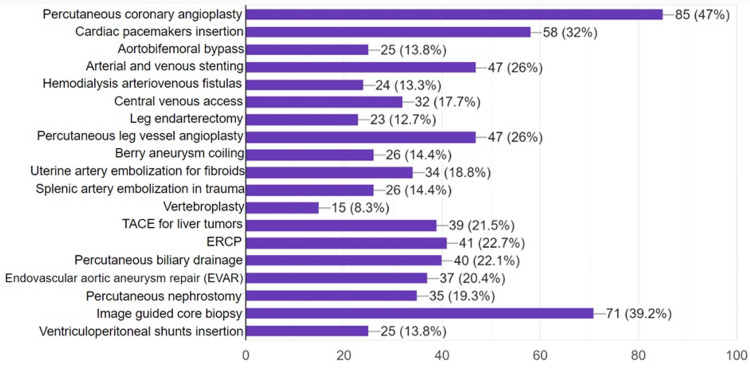
Students’ awareness of procedures routinely performed by interventional radiologists (n=181) ERCP: endoscopic retrograde cholangiopancreatography; TACE: transarterial chemoembolization Data is represented as frequencies (N) and percentages (%).

Students were asked about which procedures are routinely performed by interventional radiologists. Image-guided core biopsy (39.2%, n=71) was the most correctly answered procedure, followed by arterial & venous stenting (26%, n=47), and percutaneous leg angioplasty (26%, n=47). On the other hand, some procedures, such as percutaneous coronary angioplasty (47%), pacemaker insertion (32%), endoscopic retrograde cholangiopancreatography (22.7%), aortofemoral bypass (13.8%), ventriculoperitoneal shunting (13.8%), and endarterectomy (12.7%), were incorrectly assigned by the students to the routine IR practice. Only 34.8% (n=63) realized that IR procedures are implemented in cancer treatment. Students’ knowledge of other procedures is shown in Figure 3.

**Figure 4:**
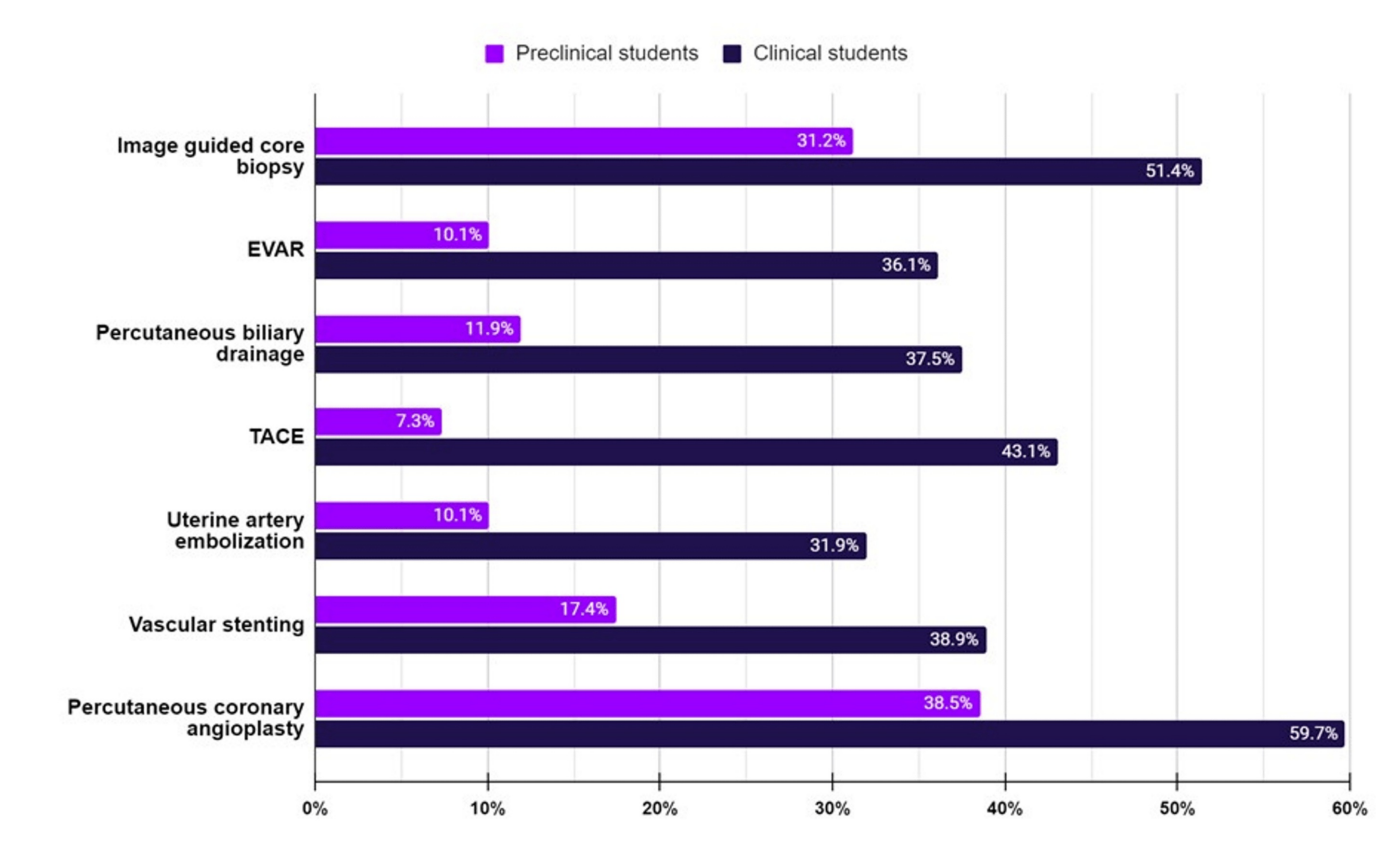
Awareness of common IR procedures among clinical (40%, n= 72) and preclinical students (60%, n= 109) EVAR: endovascular aortic repair; TACE: transarterial chemoembolization Data is represented as percentages (%).

Students in the clinical years (40%, n= 72) showed more awareness of the common IR procedures than preclinical students (60%, n= 109). However, although percutaneous coronary angioplasty is routinely performed by interventional cardiologists, 60% (n=43) of the clinical students and 39% (n=42) of the preclinical students labeled it as a routine IR procedure (Figure 4).

**Figure 5:**
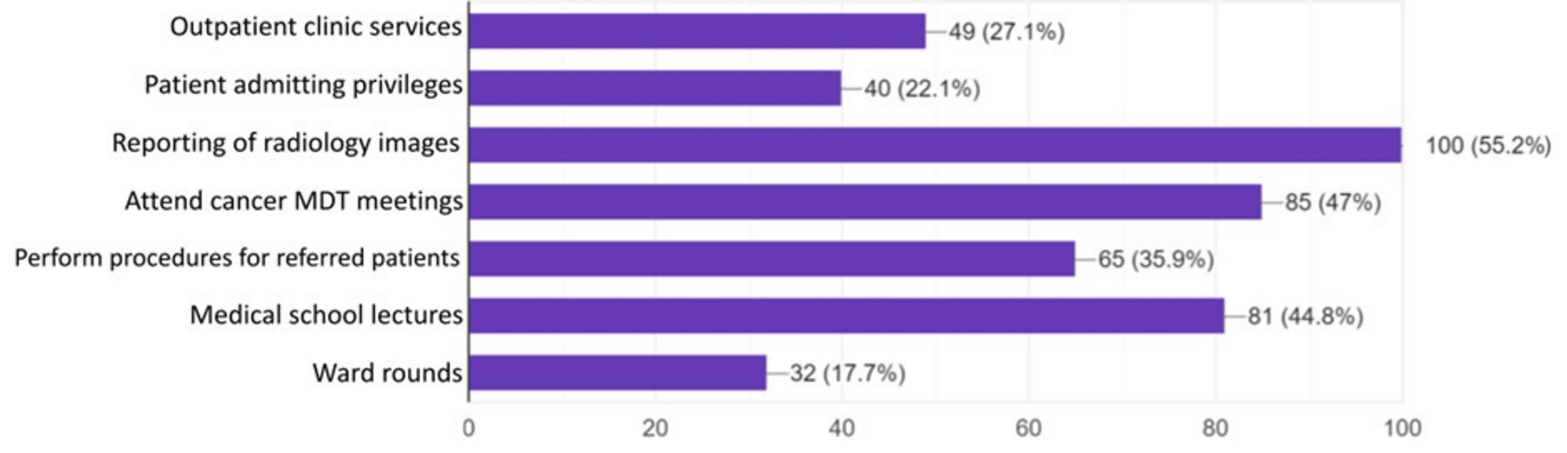
Students’ awareness of duties and roles of interventional radiologists (n=181) Data is represented as frequencies (N) and percentages (%).

Regarding the roles and duties carried out by interventional radiologists, the most known to the students were to report images (55.2%, n=100) and to attend multidisciplinary cancer meetings (47%, n=85). Participants’ awareness of other IR roles is shown in Figure 5.

**Figure 6:**
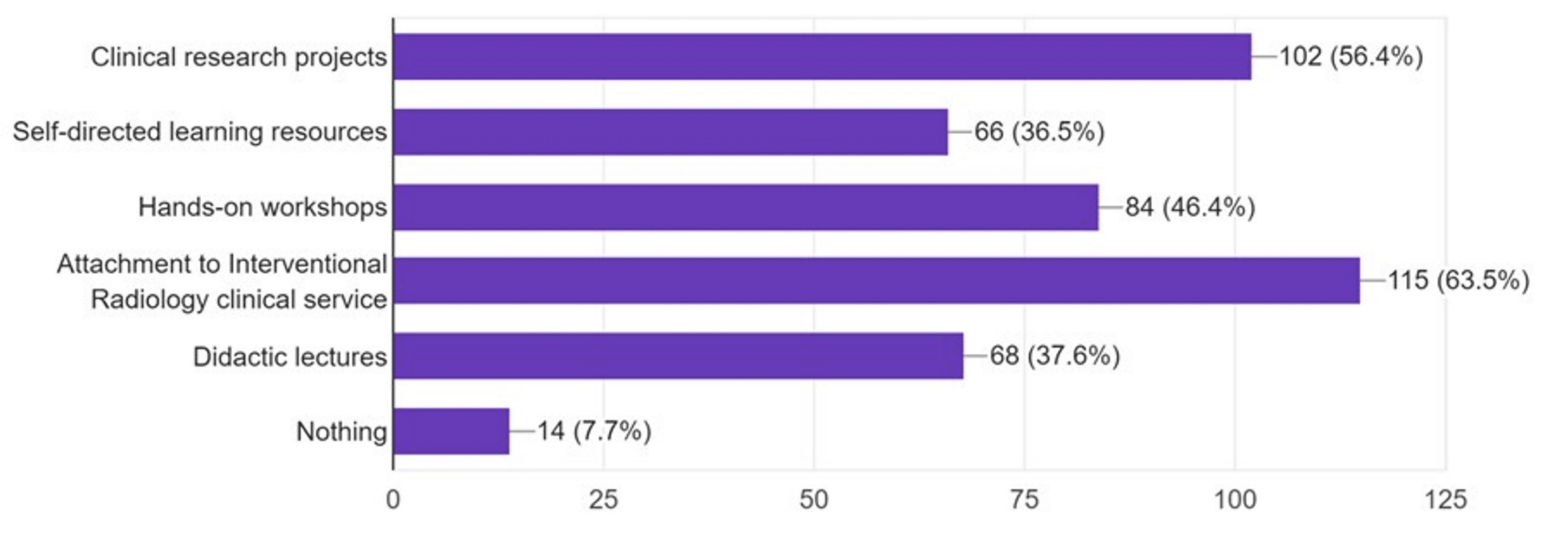
Educational activities and learning methods suggested by the participants to provide undergraduate interventional radiology education (n=181) Data is represented as frequencies (N) and percentages (%).

Most students (80.1%, n=145) were interested in learning more about IR. 64.6% of them (n=117) suggested teaching IR at the undergraduate level. They also suggested many educational activities to teach IR, including clinical attachments (63.5%, n=115) and clinical research projects (56.4%, n=102), among others (Figure 6).

According to their current knowledge base and perception of the field, only 13.8% (n=25) of the students would consider a career in IR. The majority (63.5%, n=115) needed more information to consider it, while 22.7% (n=41) showed no interest.

## Discussion

IR is a relatively new, rapidly developing field with wide applications and potential. As suggested by many international studies, there is a lack of knowledge and awareness of IR among medical students, especially in developing countries where the practice of this field is limited. This study provides subjective and objective measures of awareness of the IR specialty among medical students at the University of Khartoum, as well as an updated review of the status of IR in Sudan after the 2023 war.

Students’ knowledge of this field and other new postgraduate subspecialties is suggested to be lacking, especially among preclinical students. Early career orientation and postgraduate pathway awareness are important, allowing undergraduate students to pursue their university years well-oriented and planned. However, this should never limit undergraduate students from exploring all medical specialties and areas. The postgraduate requirements in recent years have been increasing and more demanding; to achieve higher qualification standards, students might focus on and excel in one or two specific areas, jeopardizing other disciplines, resulting in vertical building and improvement in one area rather than a broad and wide base of knowledge and skills in the different areas of medicine. IR is not part of the undergraduate curriculum at many universities, and some do not provide dedicated DR courses.

Other terms are used to refer to the new, wide, and expanding field of IR. This is potentially due to the new emergence of this field and the lack of standardization of terms used to describe the practice of the field’s activities worldwide. Students were less aware of the other names used to refer to IR. Similar findings were reported by another international study describing percutaneous surgery to be the least known term [24]. The rapid development of IR is augmented by its advantages over conventional surgeries. Select IR procedures might be more efficient, providing shorter recovery periods, less risk of complications, and lower costs [3]. Most students were able to recognize the potential decrease in the risk of complications. Despite its high value, relatively lower costs of IR procedures were noted by only 13.8% of the participants.

Numerous procedures are performed by interventional radiologists, including image-guided core biopsy, endovascular aortic repair, percutaneous biliary drainage, transarterial chemoembolization, etc. Overall, there was some confusion and a lack of knowledge regarding routine IR procedures among the students. A great confusion could be noticed regarding the awareness of percutaneous coronary angioplasty (PCA), as almost half of them incorrectly considered it as a routine IR procedure rather than a cardiology one. Consistently, in another study, final-year medical students believed that interventional radiologists routinely perform coronary angioplasty (60%), arteriovenous fistulas (47%), and arterial bypasses (30%) [23]. Vertebroplasty was the least known procedure to the students in our study (8.3%) and another study (13%) [23]. Of the students, 22.7% (n=41) considered ERCP an IR procedure rather than a gastroenterology one. In another survey, 22 out of 48 final students incorrectly assigned ERCP to surgical specialties [29]. As mentioned, in Sudan, many IR procedures are performed by vascular surgery, urology, DR, interventional cardiology, and other surgical specialties owing to the limited availability of IR services. This overlap in clinical practice may have directly affected the students’ awareness of the standard practice of these procedures and led to this confusion.

Interventional radiologists carry out multiple duties and tasks in their practice, but the perception and recognition of these roles by healthcare workers and students are inadequate. About half of the students described reporting images (55.2%) as one of the duties of interventional radiologists, while most students did not recognize ward rounds as such. Consistently, a lack of recognition of ward rounds in IR practice is also noted in other studies [6, 23].

On the other hand, most students (80.1%) were interested in learning more about IR; 64.6% suggested teaching IR at the undergraduate level. Similarly, in another study, almost all students were willing to learn more about IR and suggested its incorporation into undergraduate curricula [24]. 63.5% of students believe that IR clinical attachments can provide good education and exposure. This is in agreement with another study where most students noted that clinical attachments would improve their IR knowledge [6]. While medical students need to have basic awareness of not only IR but also other postgraduate subspecialties, the incorporation of these advanced areas into the undergraduate curricula is not an easy task. More research is required, as well as attention by medical education specialists to the quality and quantity of introduction of the advanced postgraduate subspecialties to the undergraduate level. Most participants were not considering an IR career according to their perception of the specialty. This lack of interest stems from two interplaying factors: lack of awareness and lack of exposure. More research and educational interventions are to be carried out to address these factors.

This study was, however, limited to only one medical school; a larger study from multiple schools would have allowed for a national-level comparison besides the international one. As mentioned above, many national medical schools do not provide dedicated radiology courses for undergraduate students. Another limitation of this study could be the situation posed by the recent war in Sudan, which has largely affected medical education and the practice of medicine, especially IR.

## Conclusions

IR practice in Sudan is deficient, especially after the war. Although their awareness of the field and its applications was subjectively and objectively inadequate, students showed an interest in learning more about IR and suggested its introduction to their undergraduate level. However, most students were not considering an IR career according to their current perception of the specialty. This lack of interest stems from two interplaying factors: lack of awareness and lack of exposure. While IR knowledge and exposure need to be improved, how much of IR and other advanced postgraduate subspecialties could be introduced to the undergraduate level requires more research and careful attention.

## Appendices

The online questionnaire used in the study comprised a section on participants’ characteristics (Table 2) and a section on their knowledge and awareness of IR (Table 3).

Questionnaire

**Table 2:** Section 1: Sociodemographic data of the participants.

Question	Answer Options
1. Age	☐ 17-20 ; ☐ 26-30; ☐ 21-25; ☐ =/+31
2. Sex	☐ Male ; ☐ Female
3. Select your level:	☐ Preclinical (3rd year and below); ☐ Clinical (4th year and above)

**Table 3:** Section 2: Knowledge about Interventional Radiology among the participants.

Question	Answer Options
1. Which of the following names can be used to refer to the field of Interventional Radiology? (You can choose more than one choice)	☐ Interventional Radiology; ☐ Interventionalism; ☐ Image Guided Minimally Invasive Surgery; ☐ Percutaneous Surgery; ☐ Vascular and Interventional Radiology
2. Your knowledge about Interventional Radiology is:	☐ Excellent; ☐ Good; ☐ Adequate; ☐ Poor; ☐ None
3. Select your level:	☐ Preclinical (3rd year and below); ☐ Clinical (4th year and above)
3. What are Interventional Radiologists? (You can choose more than one choice)	☐ Radiologists; ☐ Special surgeons; ☐ Vascular surgeons working with X-rays; ☐ General surgeons working with X-rays; ☐ Special radiologists who work with percutaneous techniques
4. What should be the training of an Interventional Radiologist? As a:	☐ Surgeon; ☐ Radiologist; ☐ Both; ☐ Neither
5. Which of these procedures are routinely performed by interventional radiologists? (You can choose more than one choice)	☐ Percutaneous coronary angioplasty; ☐ Cardiac pacemakers insertion; ☐ Aortobifemoral bypass; ☐ Arterial & venous stenting; ☐ Haemodialysis arteriovenous fistulas; ☐ Central venous accesses; ☐ Leg endarterectomy; ☐ Percutaneous leg vessel angioplasty; ☐ Berry aneurysm coiling; ☐ Uterine artery embolization for fibroids; ☐ Splenic artery embolization in trauma; ☐ Vertebroplasty; ☐ Trans-arterial hepatic chemo-embolization (TACE) for liver tumors; ☐ Endoscopic retrograde cholangio-pancreatography (ERCP); ☐ Percutaneous biliary drainage; ☐ Endovascular Aortic Aneurysm Repair (EVAR); ☐ Percutaneous nephrostomy; ☐ Image guided core biopsy; ☐ Ventriculo-peritoneal shunts insertion
6. Who usually performs percutaneous transluminal angioplasty (PTA)? (You can choose more than one choice)	☐ Interventional Cardiologist; ☐ Vascular surgeons; ☐ Interventional Radiologist; ☐ Others
7. Select the clinical duties and roles that are performed by an Interventional Radiologist (You can choose more than one choice)	☐ Outpatient clinic services; ☐ Patient admitting privileges; ☐ Reporting of radiology images; ☐ Attend cancer multidisciplinary team (MDT) meetings; ☐ Perform procedures for referred patients; ☐ Medical school Lectures; ☐ Ward rounds
8. Would you like to learn more about Interventional Radiology?	☐ Yes; ☐ No; ☐ Don't know
9. Would you like this subject to be taught during your medical Undergraduate training?	☐ Strongly agree; ☐ Agree; ☐ Neutral; ☐ Disagree; ☐ Strongly disagree
10. In your opinion, what could improve education about Interventional Radiology at your medical school? (You can choose more than one choice)	☐ Clinical research projects; ☐ Self-directed learning resources; ☐ Hands-on workshops; ☐ Attachment to Interventional Radiology clinical service; ☐ Didactic lectures; ☐ Nothing
11. Would you consider a career in Interventional Radiology based on your current knowledge?	☐ Yes; ☐ No; ☐ I need more information
